# Bioinformatics analysis reveals the competing endogenous RNA (ceRNA) coexpression network in the tumor microenvironment and prognostic biomarkers in soft tissue sarcomas

**DOI:** 10.1080/21655979.2021.1879566

**Published:** 2021-02-15

**Authors:** Dandan Zou, Yang Wang, Meng Wang, Bo Zhao, Fei Hu, Yanguo Li, Bingming Zhang

**Affiliations:** a Department of Radiology, The First Hospital of Qinhuangdao, Qinhuangdao, Hebei, China; b Department of MRI, The Third Hospital of Qinhuangdao, Qinhuangdao, Hebei, China; c Department of Clinical Laboratory, The First Hospital of Qinhuangdao, Qinhuangdao, Hebei, China

**Keywords:** Soft tissue sarcomas, tumor microenvironment, estimate, prognosis, ceRNA

## Abstract

Soft tissue sarcomas (STSs) are rare, heterogeneous mesenchymal neoplasias. Understanding the tumor microenvironment (TME) and identifying potential biomarkers for prognosis associated with the TME of STS might provide effective clues for immune therapy. We evaluated the immune scores and stromal scores of STS patients by using the RNA sequencing dataset from The Cancer Genome Atlas (TCGA) database and the ESTIMATE algorithm. Then, the differentially expressed mRNAs (DEGs), miRNAs (DEMs) and lncRNAs (DELs) were identified after comparing the high- and low-score groups. Next, we established a competing endogenous RNA (ceRNA) network and explored the prognostic values of biomarkers involved in the network with the help of bioinformatics analysis. High immune score was significantly associated with favorable overall survival in STS patients. A total of 328 DEGs, 18 DEMs and 67 DELs commonly regulated in the immune and stromal score groups were obtained. A ceRNA network and protein–protein interaction (PPI) network identified some hub nodes with considerable importance in the network. Kaplan–Meier survival analysis demonstrated that nine mRNAs, two miRNAs and three lncRNAs were closely associated with overall survival of STS patients. Gene set enrichment analysis (GSEA) suggested that these three lncRNAs were mainly involved in immune response-associated pathways in STS patients. Finally, the expression levels of five mRNAs (APOL1, EFEMP1, LYZ, RARRES1 and TNFAIP2) were verified, which were consistent with the results of the TCGA cohort. The results of our study confirmed the prognostic value of immune scores for STS patients. We also identified several TME-related biomarkers that might contribute to prognostic prediction and immune therapy.

## Graphical Abstract

**Figure:**
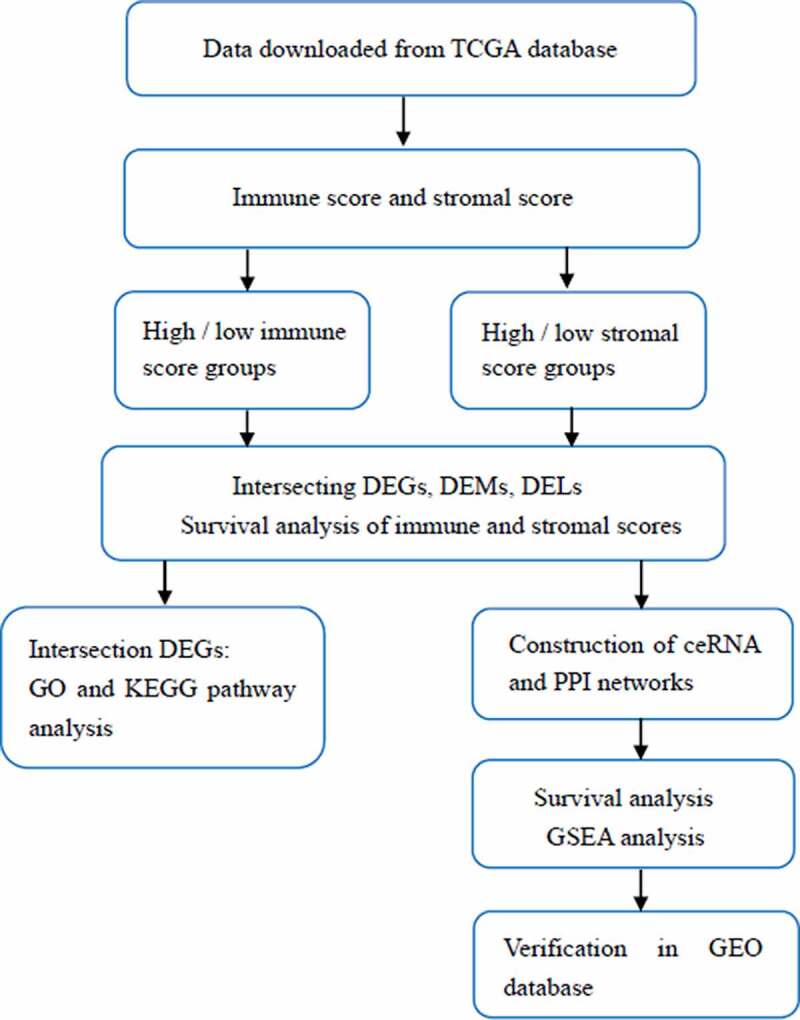

## Introduction

Soft tissue sarcomas (STSs) are a heterogeneous group of rare mesenchymal neoplasias, accounting for 1–2% of all adult malignancies [1]. It is estimated that there will be approximately 13,130 STS diagnoses and 5,350 deaths in 2020 [2]. Despite the combination of surgery resection, radiotherapy, chemotherapy, and other systemic treatment for patients with localized STS, the 5-year survival rate is only 50%-60% [3]. In addition, over 50% of patients may experience recurrence and metastasis after surgery [4].

In recent years, immunotherapeutic strategies have shown promising results in the field of cancer treatment, and the components of tumor microenvironment (TME) could significantly affect the therapeutic response in several cancer types, including STS [5–7]. One of the most successful strategies involves immune checkpoint inhibitors, such as programmed cell death-1 (PD-1) and programmed death-ligand 1 (PD-L1). The expression of PD-1 and PD-L1 is significantly correlated with CD8+ tumor infiltrating lymphocytes [8]. Their expression was also associated with clinical stage, distant metastasis, level of tumor differentiation, overall survival and event-free survival in STS patients [9]. These results suggest that PD-1 and PD-L1 are promising targets for STS patients. Thus, understanding the TME and identifying potential biomarkers associated with the TME of STS is critical to improving the efficacy of immune therapy.

As two essential components of TME, infiltrating immune and stromal cells could impact cancer prognosis [10]. The Estimation of STromal and Immune cells in Malignant Tumors using Expression data (ESTIMATE) algorithm has made it possible to predict the infiltration of immune and stromal cells in tumors by generating immune and stromal scores of each tumor sample [11]. Recently, ESTIMATE has been applied in several cancers, such as head and neck squamous cell carcinoma [12], glioblastoma [13] and acute myeloid leukemia [14], to explore potential TME-related biomarkers. Previous studies on the TME of STS have identified some immune-related genes as candidate prognostic biomarkers, which might help predict response to immunotherapy for STS [15–17]. However, the usage of ESTIMATE to explore the competing endogenous RNA (ceRNA) roles in the TME of STSs remains to be elucidated.

The hypothesis of ceRNAs was proposed as a complex posttranscriptional regulatory network in which lncRNAs, and mRNAs competed with miRNAs via miRNA response element (MRE) [18]. This competition plays a crucial role in development, progression, recurrence and prognosis of sarcomas by affecting the expression levels of various RNAs through MREs, including STS. For example, lncRNA TUG1 was reported to function as a ceRNA of miR-212-3p, and the inhibition of miR-212-3p could reverse the effect of TUG1 knockdown on osteosarcoma cell proliferation and apoptosis [19]. Runzhi Huang et al. illustrated that hsa-miR-1226-3p might play an important role in STS recurrence by regulating MUC1 and dendritic cells resting [20]. The ceRNA network, lncRNA (KCNQ1OT1)-miRNA (has-miR-29 c-3p)-mRNA (JARID2, CDK8, DNMT3A and TET1) might be a promising therapeutic target for the STS sub-cluster associated with a poor prognosis [21]. In this study, we applied the ESTIMATE algorithm to RNA sequencing data downloaded from The Cancer Genome Atlas (TCGA) database to assess the immune and stromal scores and construct a ceRNA network associated with the TME of STS. Subsequently, overall survival analysis of immune scores, stromal scores and the biomarkers involved in the ceRNA network were performed. Finally, two STS cohorts of the Gene Expression Omnibus(GEO) database were used for verification. Through these bioinformatics analyses, our study may help elucidate the TME effect on STS and provide potential targets for the immunotherapy.

## Materials and methods

### Immune and stromal score determination and differentially expressed mRNAs (DEGs), miRNAs (DEMs) and lncRNAs (DELs)

Expression profiles and basic clinical information for 217 STS samples were retrieved from TCGA (https://gdc.nci.nih.gov/). The RNA sequencing data of the GSE21122 and GSE71118 cohort were downloaded from the GEO database (https://www.ncbi.nlm.nih.gov/geo/) for validation. The immune and stromal scores were evaluated by applying the ESTIMATE algorithm using the estimate R package (R version 3.5.3) [11]. The scores were used to reflect the level of immune cell and stromal cell infiltration of tumor tissue.

According to the median immune and stromal scores, these STS samples were categorized into high- and low-score groups. Differentially expressed mRNAs (DEGs), miRNAs (DEMs) and lncRNAs (DELs) in these comparisons were filtered using the limma package of R software. The cutoff criteria were set as fold change (FC) >1.5 or <0.7 and false discovery rate (FDR) adjusted P value <0.05. Then, intersecting DEGs, DEMs and DELs of the immune and stromal score groups, shown in Venn diagrams, were selected for further analysis.

### GO and KEGG analysis of DEGs

Gene Ontology (GO) and Kyoto Encyclopedia of Genes and Genomes (KEGG) analyses of the intersecting DEGs were performed with clusterProfiler package of R software. GO terms, including biological processes (BP), molecular functions (MF), and cellular components (CC), were evaluated. P < 0.05 represents a significant difference.

### Construction of the ceRNA and protein–protein interaction (PPI) network

Miranda (http://www.microrna.org/) and TargetScan (http://www.targetscan.org/) were utilized to predict mRNA–miRNA interactions, MiRanda and PITA (https://genie.weizmann.ac.il/pubs/mir07/mir07_data.html) were used to identify lncRNA–miRNA interactions. Among them, the negatively regulated pairs that also differentially expressed were chosen for construction of the ceRNA network. In addition, the DEGs involved in the ceRNA network were modeled in a PPI network constructed using STRING (https://string-db.org/). The networks were visualized by Cytoscape software (version 3.6.1).

### Survival analysis

Based on the overall survival time of STS patients, Kaplan–Meier survival analysis was conducted to evaluate the prognostic value of immune scores, stromal scores and all biomarkers identified in the ceRNA network. Log-rank P value less than 0.05 was considered statistically significant.

### Gene set enrichment analysis (GSEA)

Samples from TCGA were divided into high and low expression groups, and GSEA was performed by using Broad Institute GSEA version 4.0 to search for KEGG pathways enriched in the highly expressed samples [22]. The enriched pathways were identified with the criteria of normal P < 0.05 and FDR <0.25.

### Statistical analysis

R packages ‘estimate, limma, clusterProfiler and survival’ were applied for statistical analyses. A two-tailed P value less than 0.05 was considered statistically significant.

### Results

This study aimed to use ESTIMATE algorithm to estimate the immune and stromal scores and construct a ceRNA network associated with the TME of STS. Then, survival analysis of immune scores, stromal scores and the biomarkers involved in the ceRNA network were performed. Finally, two STS cohorts of GEO database were used for verification. Our study may help elucidate the TME effect on STS and provide potential targets for the immunotherapy.

### Immune scores are significantly associated with overall survival of STS patients

RNA sequencing datasets of 217 STS patients from TCGA were analyzed. Pathological subtypes included 134 (61.8%) leiomyosarcomas, 42 (19.4%) dedifferentiated liposarcomas, 26 (12.0%) undifferentiated pleomorphic sarcomas, nine (4.1%) myxofibrosarcomas, three (1.4%) synovial sarcomas, two (0.9%) desmoid tumors, and one (0.4%) malignant peripheral nerve sheath tumor. The age ranged from 20 to 90 years, 45.2% were male and 54.8% were female. Other clinical information can be found in Supplementary Table S1. With the ESTIMATE algorithm, we found that the immune scores for the patients ranged from −1750.11 to 3630.51, and the stromal scores ranged from −1384.46 to 2518.94 (Table S2).

**Figure 1.**
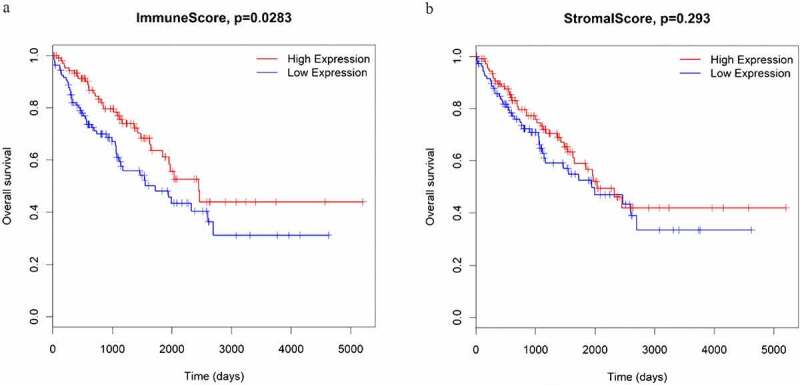
The associations between immune scores (a) and stromal scores (b) and overall survival in STS patients

Subsequently, to determine the relationship between immune and stromal scores and survival in STS samples, Kaplan–Meier survival analyses were performed. The results showed that STS patients with high immune scores had significantly longer overall survival than those with low immune scores (P = 0.0283; Figure 1(a)). Meanwhile, STS patients with high stromal scores also had more favorable outcomes than those with low stromal scores, although the difference was not statistically significant (P = 0.293; Figure 1(b)).

### DEGs, DEMs and DELs obtained based on immune and stromal scores

**Figure 2.**
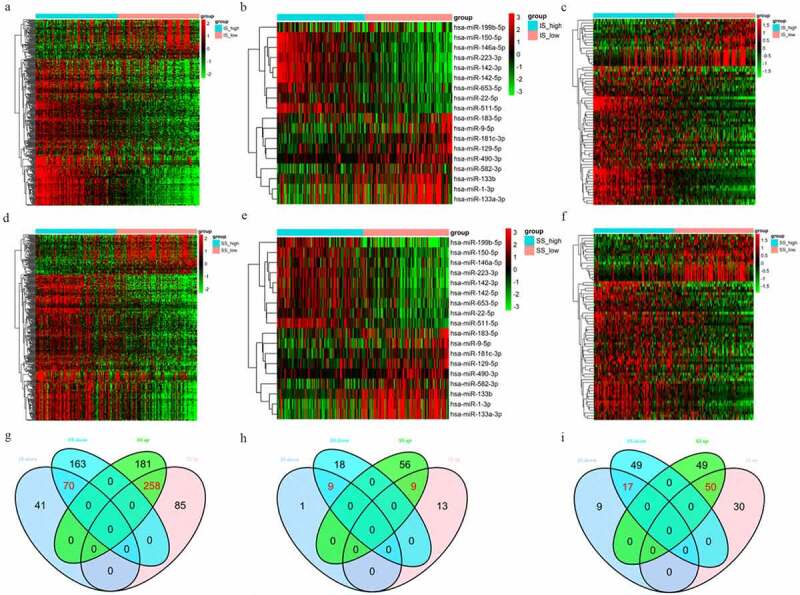
(a-c) Heatmaps of DEGs, DEMs and DELs in the high vs. low immune score groups. (d-f) Heatmaps of DEGs, DEMs and DELs in the high vs. low stromal score groups. Green represents high expression, and red represents low expression. (g-i) Venn diagrams showing the number of DEGs, DEMs and DELs common to the immune and stromal score groups

In the high immune score vs. low immune score group, 454 DEGs, 32 DEMs and 106 DELs were identified. In the high stromal scores vs. low stromal scores group, 672 DEGs, 92 DEMs and 165 DELs were identified (Figure 2(a-f)). A total of 328 DEGs (258 upregulated and 70 downregulated), 18 DEMs (9 upregulated and 9 downregulated) and 67 DELs (50 upregulated and 17 downregulated) that were commonly regulated in these two groups were obtained through Venn diagramming (Figure 2(g-i)).

### GO and KEGG analysis of DEGs

**Figure 3.**
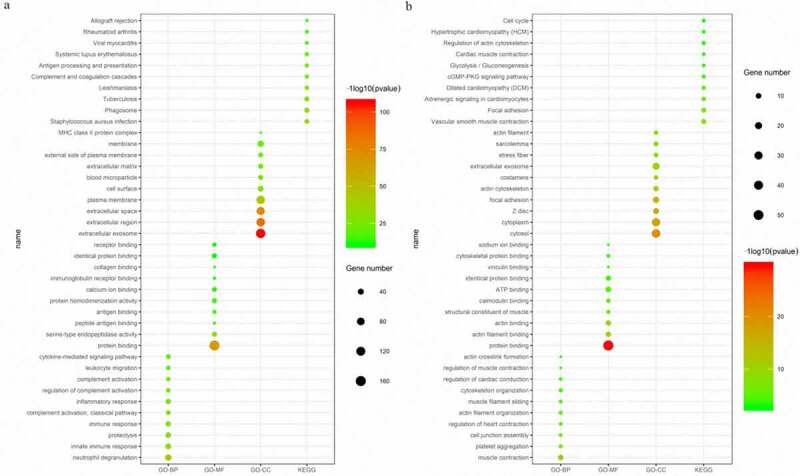
The top 10 BP, MF, CC and pathway terms for the upregulated (a) and downregulated (b) DEGs

The 328 commonly regulated DEGs were further analyzed to explore their potential functions. The top GO terms among the upregulated DEGs included neutrophil degranulation and innate immune response in BP; protein binding and serine-type endopeptidase activity in MF; and extracellular exosome and extracellular region in CC. The downregulated GO terms included muscle contraction and platelet aggregation in BP; protein binding and actin filament binding in MF; and cytosol and cytoplasm in CC (Figure 3(a, b)).

Additionally, for the KEGG pathways, the upregulated DEGs were significantly enriched in *Staphylococcus aureus* infection, phagosome and tuberculosis, and the downregulated DEGs were enriched in vascular smooth muscle contraction, focal adhesion and adrenergic signaling in cardiomyocytes (Figure 3(a, b)). Furthermore, some of the GO terms and pathways were closely associated with immune processes.

### Construction of ceRNA and PPI Network

**Figure 4.**
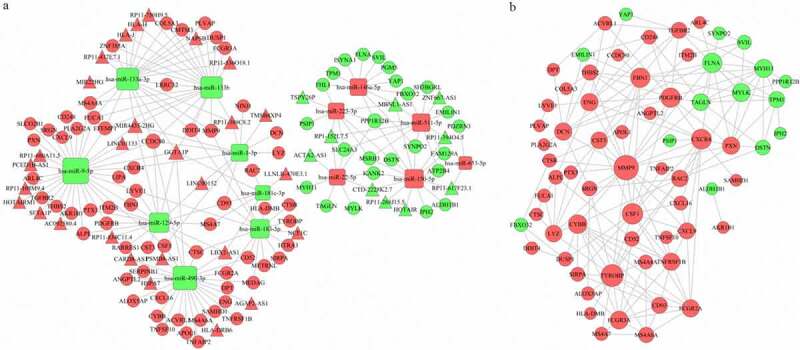
The ceRNA network (a) and the PPI network (b). Red indicates upregulation, and green indicates downregulation. The circle nodes represent DEGs, the rectangle nodes represent DEMs, and the triangle nodes represent DELs

The 18 DEMs were used to predict their target mRNAs and lncRNAs. A total of 347 mRNAs and 260 lncRNAs that targeted with these miRNAs were obtained (Table S3, S4). Then, we compared the resulting predicted negatively regulated pairs with the DEGs and DELs, and 89 DEGs, 14 DEMs and 38 DELs were finally used to construct the ceRNA network. The network consisted of 142 nodes and 424 edges, and the degrees of the top 5 nodes (hsa-miR-9-5p, hsa-miR-490-3p, hsa-miR-133a-3p, hsa-miR-133b and hsa-miR-129-5p) were 32, 27, 23, 22 and 17, respectively (Figure 4(a)).

Then, the PPI network, which consisted of 67 nodes (52 upregulated and 15 downregulated genes) and 180 edges, was constructed to show the interactions between the DEGs in the ceRNA network. The top 10 genes with the most interactions (MMP9, TYROBP, CSF1, CXCR4, FBN1, FLNA, PDGFRB, CYBB, FCGR3A and MYH11) were considered hub genes based on their degree of importance (Figure 4(b)). Among them, MMP9 and FLNA were the upregulated and downregulated genes with highest degree (20 and 12), respectively. This means that they have the most connections with other genes and play important roles in the network.

### Survival analysis

**Figure 5.**
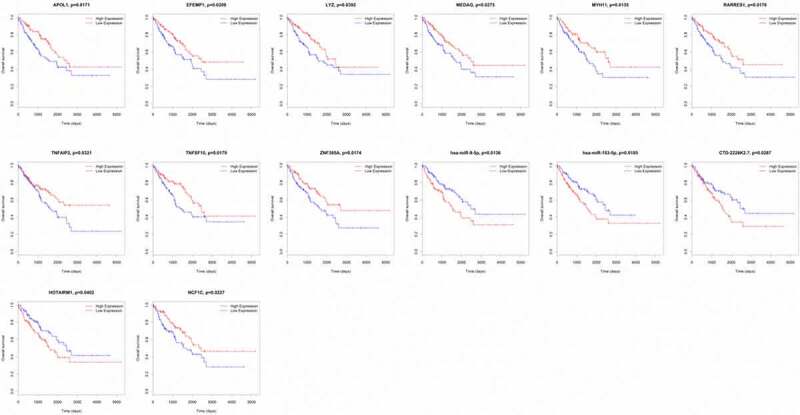
Kaplan–Meier survival curves of APOL1, EFEMP1, LYZ, MEDAG, MYH11, RARRES1, TNFAIP2, TNFSF10, ZNF385A, hsa-miR-9-5p, hsa-miR-183-5p, CTD-2228K2.7, HOTAIRM1 and NCF1C

We analyzed the associations between the 89 DEGs, 14 DEMs and 38 DELs in the ceRNA network and the overall survival of STS patients. Nine mRNAs (APOL1, EFEMP1, LYZ, MEDAG, MYH11, RARRES1, TNFAIP2, TNFSF10 and ZNF385A) among 89 DEGs were closely related to the overall survival of STS patients. The high expression of all nine of these mRNAs was associated with a high survival rate in STS patients. We also observed that the low expression of two miRNAs (hsa-miR-9-5p and hsa-miR-183-5p) among the 14 DEMs was related to favorable survival outcomes. In addition, three lncRNAs (CTD-2228K2.7, HOTAIRM1 and NCF1C) were closely associated with the overall survival of STS patients. For CTD-2228K2.7 and HOTAIRM1, low expression was related to a high overall survival rate in STS patients. For NCF1C, high expression was correlated with longer overall survival time (Figure 5).

### GSEA

We performed GSEA to identify the pathways associated with the three survival-associated lncRNAs (CTD-2228K2.7, HOTAIRM1 and NCF1C). The normalized enrichment score (NES) was used to sort the most significantly positively and negatively enriched pathways. As shown in Table 1, CTD-2228K2.7 was negatively correlated with systemic lupus erythematosus, leishmania infection and hematopoietic cell lineage; however, no pathways were significantly enriched in the samples with high expression levels of CTD-2228K2.7. Interestingly, the top five most positive (lysosome, n glycan biosynthesis, vibrio cholerae infection, amino sugar and nucleotide sugar metabolism and proteasome) and most negative pathways (propanoate metabolism, vascular smooth muscle contraction, inositol phosphate metabolism, lysine degradation and valine leucine and isoleucine degradation) were commonly enriched in the high and low HOTAIRM1 and NCF1C expression groups. This suggests that CTD-2228K2.7, HOTAIRM1 and NCF1C may contribute to the TME of STS patients by functioning in several immune-associated signaling pathways.

**Table 1.** GSEA analyses of CTD-2228K2.7, HOTAIRM1 and NCF1C

CTD-2228K2.7	KEGG name	NES	NOM p-val	FDR q-val
	systemic lupus erythematosus	−1.9380207	0.005928854	0.0488711
	leishmania infection	−1.9338785	0.012096774	0.0346511
	hematopoietic cell lineage	−1.9176999	0.004	0.0313945
	type i diabetes mellitus	−1.9036341	0.007984032	0.0292524
	natural killer cell mediated cytotoxicity	−1.8832018	0.01622718	0.0298049
HOTAIRM1				
	lysosome	2.0215623	0	0.0222536
	n glycan biosynthesis	1.9878095	0	0.017478
	vibrio cholerae infection	1.880461	0	0.0592255
	amino sugar and nucleotide sugar metabolism	1.8698859	0.00409836	0.0526439
	proteasome	1.8244344	0.001968504	0.0674427
	propanoate metabolism	−1.9495336	0	0.0645897
	vascular smooth muscle contraction	−1.9222941	0	0.0507997
	inositol phosphate metabolism	−1.8443166	0.005791506	0.0843508
	lysine degradation	−1.8133465	0.008230452	0.08976
	valine leucine and isoleucine degradation	−1.7961998	0.004081633	0.0849993
NCF1C	lysosome	2.0150259	0	0.0132608
	n glycan biosynthesis	1.923236	0.001976285	0.0407005
	amino sugar and nucleotide sugar metabolism	1.909916	0	0.0345147
	vibrio cholerae infection	1.9047865	0	0.028172
	proteasome	1.8770373	0.005535055	0.0341476
	propanoate metabolism	−1.959569	0	0.0707392
	vascular smooth muscle contraction	−1.9155267	0.00409836	0.0590163
	valine leucine and isoleucine degradation	−1.8557318	0	0.0803684
	inositol phosphate metabolism	−1.8172265	0.008064516	0.0900969
	lysine degradation	−1.8166823	0.002016129	0.0722303

### Further validation using two additional independent cohorts

**Figure 6.**
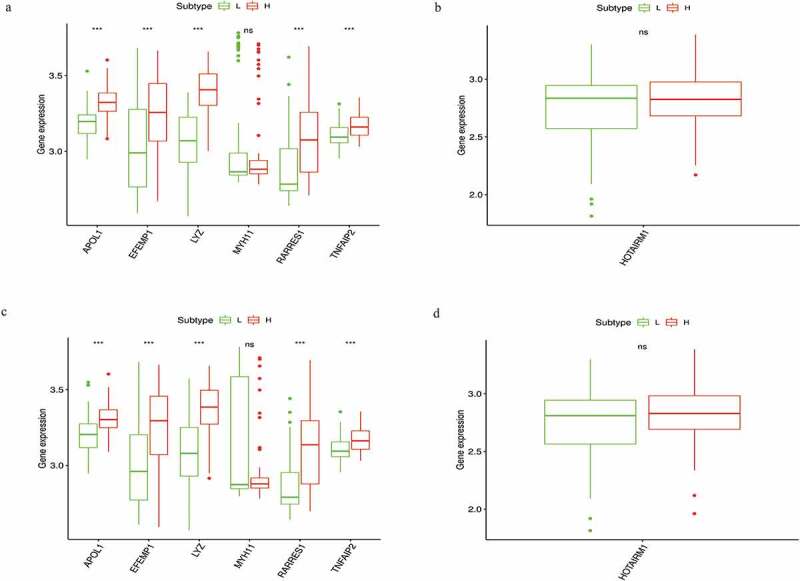
Verification of six mRNAs (APOL1, EFEMP1, LYZ, MYH11, RARRES1, and TNFAIP2) and one lncRNA (HOTAIRM1) of high vs. low immune score groups (a, b) and high vs. low stromal score groups (c,d) in GEO database

To verify whether these prognostic biomarkers we identified were also of prognostic significance in GEO database, we downloaded two STS cohorts for analysis. GSE21122 with 149 STS cases and GSE71118 with 312 cases were chosen for mRNA and lncRNA verification, respectively. While for lacking sufficient RNA sequencing data and survival information, only the differential expression analysis of six mRNAs (APOL1, EFEMP1, LYZ, MYH11, RARRES1 and TNFAIP2) and one lncRNA (HOTAIRM1) between the high- and low-score groups were successfully conducted. The results indicated that the expression levels of five mRNAs (APOL1, EFEMP1, LYZ, RARRES1 and TNFAIP2) were also upregulated in the two comparisons (all P < 0.05), which was consistent with the results of the TCGA cohort (Figure 6). Unfortunately, for the mRNA MYH11 and lncRNA HOTAIRM1, we could not find significant difference between the two groups.

## Discussion

The current study was the first to use ESTIMATE algorithm to investigate the ceRNA network associated with the TME of STSs based on TCGA. We found that STS patients with high immune scores had longer overall survival, suggesting that TME was closely correlated with clinical outcomes. This result was also similar to a previous study indicating that high immune scores have favorable outcomes in osteosarcoma patients [23]. That may be because higher immune scores suggested an enhanced immune system and function, which could increase the antitumor immunity of TME and contribute to controlling and eliminating the tumor [24,25]. Unfortunately, for the association between stromal scores and overall survival of STS samples, we failed to find any significant difference.

Subsequently, we identified 328 DEGs, 18 DEMs and 67 DELs that were common to both the high- and low-score groups. GO and pathway analyses showed that many of the DEGs participate in immune processes. Next, we identified hsa-miR-9-5p, hsa-miR-490-3p, hsa-miR-133a-3p, hsa-miR-133b and hsa-miR-129-5p as the top five nodes in the ceRNA network and MMP9, TYROBP, CSF1, CXCR4, FBN1, FLNA, PDGFRB, CYBB, FCGR3A and MYH11 as the top 10 genes in the PPI network. These results indicated that these genes might have more significant functions in the networks. We performed overall survival analysis for the 89 DEGs, 14 DEMs and 38 DELs in the ceRNA network and found that 9 mRNAs (APOL1, EFEMP1, LYZ, MEDAG, MYH11, RARRES1, TNFAIP2, TNFSF10 and ZNF385A), 2 miRNAs (hsa-miR-9-5p and hsa-miR-183-5p) and 3 lncRNAs (CTD-2228K2.7, HOTAIRM1 and NCF1C) were closely associated with overall survival in STS patients. Then, GSEA analyses of the three survival-associated lncRNAs identified several immune response-related pathways. Finally, we tried to validate these prognostic biomarkers in GEO database, while for lacking relevant RNA sequencing and follow-up data, we only successfully validated that the expression level of five mRNAs (APOL1, EFEMP1, LYZ, RARRES1 and TNFAIP2), which were consistent with the results of the TCGA cohort.

APOL1 is a novel BH3-only protein, and its overexpression could induce autophagy and autophagy-associated cell death in several types of cancer cells [26,27]. APOL1 was found to be overexpressed in pancreatic cancer, lung adenocarcinoma and papillary thyroid carcinomas compared to matched normal tissues, and it was found to have prognostic value for pancreatic cancer and lung adenocarcinoma [28–30]. EFEMP1 can be found in different human tissues and is a member of the fibulin family of extracellular glycoproteins [31]. High EFEMP1 expression helps enhance tumor growth in pancreatic carcinoma cells by binding the EGF receptor and activating the MAPK and Akt pathways [32]. Additionally, EFEMP1 could promote the migration and invasion of osteosarcoma via MMP-2 with induction by AEG-1 via the NF-κB signaling pathway, and EFEMP1 was also reported to be an indicator of poor prognosis in osteosarcoma [33]. LYZ encodes human lysozyme and acts as a macrophage marker, and its expression levels positively correlate with the numbers of CD68+ pSTAT1+ macrophages [34]. In addition, LYZ could interact with CD34+ cells and neutrophils, which may predict an increased risk of thrombosis in essential thrombocythemia patients [35]. RARRES1 and TNFAIP2 have been commonly investigated in many cancers. RARRES1 may induce autophagy in prostate cancer and cervical cancer cells [36,37]. A recent study confirmed that RARRES1 contributed to the regulation of dendritic cells and acted as a novel immune-related biomarker for glioblastoma [38]. TNFAIP2 is abundant in immune cells such as myelomonocytic cells, endothelial cells, peripheral blood monocytes, dendritic cells, intestinal M cells, and macrophages [39–41]. It plays essential roles in inflammation, cell proliferation, angiogenesis, migration, and membrane nanotube formation [42].

There are some limitations in the current study. Firstly, because of lacking RNA sequencing data of STS cohorts, the expression validity of survival-associated biomarkers was partially done. The expression of MEDAG, TNFSF10, ZNF385A, hsa-miR-9-5p, hsa-miR-183-5p, CTD-2228K2.7, and NCF1C could not be performed for the verification of expression levels. Secondly, although we also failed to verify the prognostic significance of all the 14 survival-associated biomarkers for the lack of outcome data, it did not mean that these biomarkers were of no prognostic value. We anticipate that sufficient evidence will be available in the near future for us to validate the conclusions. Thirdly, our study may provide new TME-related biomarkers to predict STS prognosis, but we did not perform the individual marker analysis for their association with immune cells and their potential roles in the precise mechanism of the TME of STS. Thus, further studies including clinical trials are needed to further improve and verify our results.

## Conclusions

Taken together, the findings in our study confirmed the prognostic value of immune scores for STS patients, and we found several TME-related biomarkers (APOL1, EFEMP1, LYZ, RARRES1 and TNFAIP2) that might contribute to prognostic prediction and help improve the efficacy of immune therapy.

## Supplementary Material

Supplemental Material

## Data Availability

The datasets of this article were generated from the TCGA and GEO database.

## Highlights

Soft tissue sarcomas; tumor microenvironment; ESTIMATE; prognosis; ceRNA
