# Supplementary figures and images for: Bioinformatics analysis reveals the competing endogenous RNA (ceRNA) coexpression network in the tumor microenvironment and prognostic biomarkers in soft tissue sarcomas
Source: Bioengineered. 2021 Feb 15;12(1):662–72. doi: 10.1080/21655979.2021.1879566 (PMC8806339; doi:10.1080/21655979.2021.1879566)

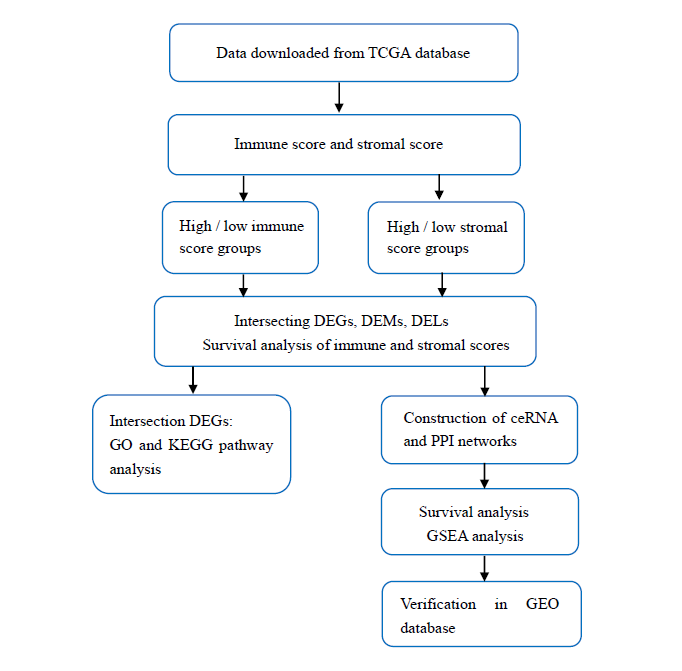

Supplement: Supplemental Material [file KBIE_A_1879566_SM9997.zip › Supplementary information/GraphicalAbstract.png]
